# Early vascular endothelial complications after hematopoietic cell transplantation: Role of the endotheliopathy in biomarkers and target therapies development

**DOI:** 10.3389/fimmu.2022.1050994

**Published:** 2022-11-21

**Authors:** Ana Belén Moreno-Castaño, María Queralt Salas, Marta Palomo, Julia Martinez-Sanchez, Montserrat Rovira, Francesc Fernández-Avilés, Carmen Martínez, Joan Cid, Pedro Castro, Gines Escolar, Enric Carreras, Maribel Diaz-Ricart

**Affiliations:** ^1^ Hemostasis and Erythropathology Laboratory, Hematopathology, Pathology Department, Centre de Diagnòstic Biomèdic (CDB), Hospital Clínic, Barcelona, Spain; ^2^ Clínic, Institut Josep Carreras, Barcelona, Spain; ^3^ Institut d’Investigacions Biomèdiques August Pi i Sunyer (IDIBAPS), University of Barcelona, Barcelona, Spain; ^4^ Hematology Department, Bone Marrow Transplantation Unit, Institut Clínic de Malalties Hemato-Oncològiques (ICMHO), Hospital Clínic, Barcelona, Spain; ^5^ Campus Clinic, Hospital Clinic de Barcelona, Barcelona, Spain; ^6^ Apheresis & Cellular Therapy Unit, Department of Hemotherapy and Hemostasis, Institut Clínic de Malalties Hemato-Oncològiques (ICMHO), Hospital Clínic de Barcelona, Barcelona, Spain; ^7^ Medical Intensive Care Unit, Hospital Clínic de Barcelona, Universitat de Barcelona, Barcelona, Spain

**Keywords:** endothelial, biomarkers, early HCT complications, laboratory diagnosis, prognosis assessment, response assessment, endothelium as therapeutic target

## Abstract

This work aims to review the role of endothelial dysfunction underlying the main complications appearing early after autologous and allogeneic hematopoietic cell transplantation (HCT). The endothelial damage as the pathophysiological substrate of sinusoidal obstruction syndrome (SOS) is well established. However, there is growing evidence of the involvement of endothelial dysfunction in other complications, such as acute graft-versus-host disease (aGVHD) and transplant-associated thrombotic microangiopathy (TA-TMAs). Moreover, HCT-related endotheliopathy is not only limited to the HCT setting, as there is increasing evidence of its implication in complications derived from other cellular therapies. We also review the incidence and the risk factors of the main HCT complications and the biological evidence of the endothelial involvement and other linked pathways in their development. In addition, we cover the state of the art regarding the potential use of the biomarkers of endotheliopathy in the prediction, the early diagnosis, and the follow-up of the HCT complications and summarize current knowledge points to the endothelium and the other linked pathways described as potential targets for the prevention and treatment of HCT-complications. Lastly, the endothelium-focused therapeutic strategies that are emerging and might have a potential impact on the survival and quality of life of post-HCT-patients are additionally reviewed.

## Introduction

Hematopoietic-cell transplantation (HCT) has been used to re-establish marrow and immune function in patients with inherited or acquired hematopoietic disorders, whether benign or neoplastic, including those of the immune system, and as enzyme replacement in metabolic disorders. HCT is additionally a strategy to support treatments that contain high-dose chemotherapy for which hematologic toxicity would otherwise limit the drug administration (1, 2). During the last two decades, allogeneic (allo) and autologous (auto) HCTs have evolved into a more effective and safe procedure secondary to the refinements on conditioning regimens, donor selection, graft-versus-host disease (GvHD) prophylaxis, and supportive care (3, 4). However, despite these well-documented improvements and its curative potential, HCT remains associated with non-negligible rates of morbidity, mortality, and a relevant impact on patient’s quality of life (5).

Early HCT-related complications, including (in chronological order) sinusoidal obstruction syndrome (SOS), engraftment syndrome (ES), capillary leak syndrome (CLS), transplant-associated thrombotic microangiopathy (TA-TMA), acute graft-versus-host disease (aGvHD), and vascular idiopathic pneumonia syndrome (vascular-IPS) –including diffuse alveolar hemorrhage (DAH), are clinically relevant events with a recognized common origin in endothelial cell (EC) activation that can potentially evolve into a non-reversible multiorgan dysfunction (MOD) (6). Therefore, a better understanding of these early post-transplant complications and their association with the endothelium is essential to establishing effective preventive and therapeutic strategies.

This review provides information about the incidence, clinical features, and treatment of early post-transplant endothelium-related complications by discussing the endothelium’s role in the pathogenesis and treatment of these complications. Moreover, the present review summarizes the potential benefit of using biomarkers of endothelial damage for the diagnosis and monitoring of vascular post-transplant endothelial complications.

## Epidemiology of HCT early complications of endothelial origin: Incidence and risk factors

There is increasing evidence that endothelial dysfunction is involved in a group of early and potentially life-threatening post-HCT endothelial complications, such as SOS, ES, CLS, TA-TMA, aGvHD, or IPS/DAH. These events generally appear during the first 100 days after the stem cell infusion. Their diagnosis is usually based on the presence of medical signs and symptoms, and all of them seem to begin at the capillary level and result from an endothelial dysfunction occasioned by the administration of chemotherapy, calcineurin inhibitors, granulocyte-colony stimulating factor (G-CSF), infections, and allogeneic-derived reactivity (7–9). Below we detail the incidence and risk factors of the most relevant complications of endothelial origin occurring during the early post-HCT period.

### Sinusoidal obstruction syndrome

SOS (formerly known as veno-occlusive disease or VOD) is a clinical and potentially life-threatening syndrome occurring after HCT, chemotherapy regimens alone, and, less commonly, after high doses of radiation or liver transplantation (10). Other well-known risk factors for SOS are older age, female gender, previous hepatic disease, pre-HCT iron overload, previous treatment with gentuzumab/inotuzumab-ozogamicin, genetic factors (11), the underlying disease, myeloablative regimens –especially those containing busulfan or fludarabine- and GvHD prophylaxis based on the combination of calcineurin inhibitors and sirolimus (12). The reported incidence of post-transplant SOS is estimated at around 5-13%, although it can reach 60%, according to transplant settings, particularly in high-risk pediatric populations, considering that the presence of several risk factors might have a summatory deleterious effect (12–15). Clinical manifestations include hepatomegaly, right upper quadrant pain, ascites, weight gain, and jaundice, although anicteric forms may occur, especially among the pediatric population (14). The diagnostic criteria of SOS slightly differ in adult and pediatric cohorts (16, 17). However, they are both based on clinical and analytical parameters, whereas transjugular hemodynamic studies or liver biopsies remain complimentary tests. SOS can significantly affect transplant outcomes, as it can evolve into a MOD characterized by pleural effusion, pulmonary infiltrates and hypoxia, renal failure, and confusion or encephalopathy. This progression is associated with a very high mortality rate, exceeding 80% in severe forms (18, 19). Nevertheless, early interventions have been correlated to a survival benefit, enhancing the importance of prevention and early diagnosis.

### Engraftment syndrome

ES is a clinical syndrome that can occur during neutrophil engraftment in patients undergoing autologous and allo-HCT. The reported incidences of ES range from 5 to 20% in autologous HCT (20, 21), and from 1% to 15% after allogeneic HCT (22, 23) respectively. Risk factors for ES are female gender, the lack of intense chemotherapy-based schemes previous to the HCT (p.e induction treatments in myeloma patients (24)) and the use of G-CSF for the peripheral blood stem cells mobilization or neutrophil recovery. Although different criteria have been defined for diagnosing ES (25), the ones proposed by Maiolino et al. seem to be the most sensitive (20). Those consist of the presence of non-infectious fever plus any of the following: erythroderma involving ≥25% body surface area not attributed to medication, non-cardiogenic pulmonary edema, or diarrhea (21). First-line treatment for ES generally is based on high-dose corticosteroids, leading to a rapid clinical response in most cases. However, ES has been associated with a higher risk of non-relapse mortality and shorter overall survival (22, 23).

### Capillary leak syndrome

CLS is a rare but potentially life-threatening complication after HCT, characterized by a generalized abnormal accumulation of fluids and macromolecules in the extravascular space leading to anasarca, hemoconcentration, severe hypotension, and, ultimately, vascular collapse and shock. CLS was postulated as one endothelial complication of HCT by extrapolation of the biological findings observed in the idiopathic systemic capillary leak syndrome or Clarkson disease (26–28). Nevertheless, the clinical presentation of CLS is unspecific and shared with most of the complications reviewed here, raising doubt about whether CLS should be considered an independent entity per se. Allo-HCT, the use of intensive doses of chemotherapy or radiation, the selection of mismatched or matched unrelated donors, and the use of G-CSF have been identified as predictors for the development of CLS after stem cell infusion. The incidence of pure CLS after allo-HCT is extremely low (29, 30). Limited data have been reported regarding the outcome of CLS and its impact on post-transplant survival. However, CLS has been consistently associated with a high mortality rate secondary to the directly induced endothelial hyper-permeability and endothelial barrier breakdown induced by this post-transplant complication (31).

### Transplant-associated thrombotic microangiopathy

TA-TMA results from the accumulation of micro-thrombi occluding the microcirculation leading to ischemic organ dysfunction, especially in the renal, intestinal, and neurological vascular beds (32). The reported TA-TMA incidence ranges from 10 to 35% in the literature and is more prevalent after allo-HCT. Moreover, the usual range time to onset of TA-TMA is generally from day 20 to 90 after the stem cell infusion (33). Risk factors include older age, female gender, HCT from unrelated donors, the use of myeloablative conditioning regimens or total body irradiation, and the diagnosis of viral or fungal infections or GvHD. The gold standard technique to confirm TA-TMA diagnosis would be performing a biopsy of the affected organ. However, it is often omitted as it has been associated with bleeding complications. Considering this limitation, different diagnostic criteria have been defined over the last decades (34–37). These criteria are based on clinical and analytical parameters, and, especially for adults, there is not yet a consensus about which ones should be used. In 2014, Jodele et al. defined the following diagnostic criteria for TA-TMA for pediatric patients undergoing HCT: acute elevation of LDH, proteinuria >30 mg/dL, anemia, thrombocytopenia, the presence of schistocytes, and hypertension (37, 38). These diagnostic criteria could potentially be extrapolated to the adult population as they are the most realistic and feasible for diagnosing this complication. The reported mortality rates in patients with clinically relevant TA-TMA have been up to 75%, partially due to the irreversible organ damage caused by delayed diagnosis on some occasions (37, 39). The use of eculizumab, a monoclonal antibody against the fraction C5 of the complement system, has improved survival in patients with severe forms of this complication, although mortality rates in treated patients still exceed 30% (40, 41).

### Graft-versus-host disease

Acute and chronic GvHD is one of the principal non-relapse complications after allo-HCT, which still causes substantial morbidity and mortality despite significant advances in treatment and supportive care (42, 43), and the prevention of GvHD, is, therefore critical to the success of allo- HCT. The main risk factor for the development of acute and chronic GvHD is the HLA disparity. Furthermore, increased age of both the recipient and the donor, gender disparity, multiparous female donors, high-intensity conditioning regimens, the infusion of peripheral blood stem cell grafts -opposed to those from bone marrow-, and the use of ineffective GvHD prophylaxis are known to be additional predictors for the development of GvHD (42, 44). The diagnosis for this complication is based on clinical features, patient symptoms, laboratory values, and in most cases, histological confirmation. Systemic steroids alone or combined with additional immunosuppressant drugs continue to be the first line of treatment for clinically relevant GvHD. Nevertheless, novel and exciting prophylaxis and therapies are being investigated, including targeting early events in GvHD pathogenesis, such as interactions between tissue damage-associated antigens and T-cells, endothelial toxicity, and T-cell trafficking (44, 45).

### Vascular idiopathic pneumonia syndrome/diffuse alveolar hemorrhage

Idiopathic pneumonia syndromes (IPS) are early non-infectious complications after HCT causing acute lung dysfunction. IPS encompasses different entities sub-classified depending on the pulmonary area affected (parenchyma, vascular endothelium, or airway epithelium) (46). Among vascular-IPS, diffuse alveolar hemorrhage (DAH) is developed in a small proportion of patients (2-14%) in both, the autologous and the allogeneic settings (47) and is characterized by the progressive bloodier return of the bronchoalveolar lavage fluid, in at least three segmental bronchi, indicating the presence of blood in the alveoli (48). Different risk factors for IPS have been identified: older recipient’s age, malignancy other than leukemia, HLA mismatch, high–intensity conditioning regimens, and the presence of concomitant acute GvHD. Diagnostic clinical criteria for Vascular-IPS include signs and symptoms of pneumonia, multilobar radiographic infiltrates, abnormal pulmonary function, and the absence of infectious etiology or other causes for fluid overload which could justify the syndrome (49, 50). The first line of treatment includes high doses of steroid therapy. However, despite the prompt start of treatment, IPS-related mortality rates are very high, ranging from 60% to 80%, and superior to 95% in patients requiring mechanical ventilation (46, 49). Specifically, overall mortality in patients presenting DAH is also very high, although it seems less dramatic when it appears early after HC (51).

## The role of endotheliopathy and other linked pathways in the development of HCT early complications

Endothelial cells (EC) tightly regulate the vascular homeostatic balance by upholding an anti-inflammatory and anti-thrombotic state to preserve proper blood circulation. Different innate and adaptative immune reactions and pathogen-associated molecular patterns in infections, together with toxic agents such as immunosuppressant medications or chemotherapies and radiation used as part of the preparative regimens have been identified as noxa towards the endothelium (52–63). Moreover, the HCT process *per se* has been demonstrated to induce endothelium dysregulation leading to a hypercoagulable state (64) by incrementing the levels of pro-coagulant molecules and decreasing the ones of the main natural-anticoagulant molecules, among other mechanisms (65, 66).

**Figure 1:**
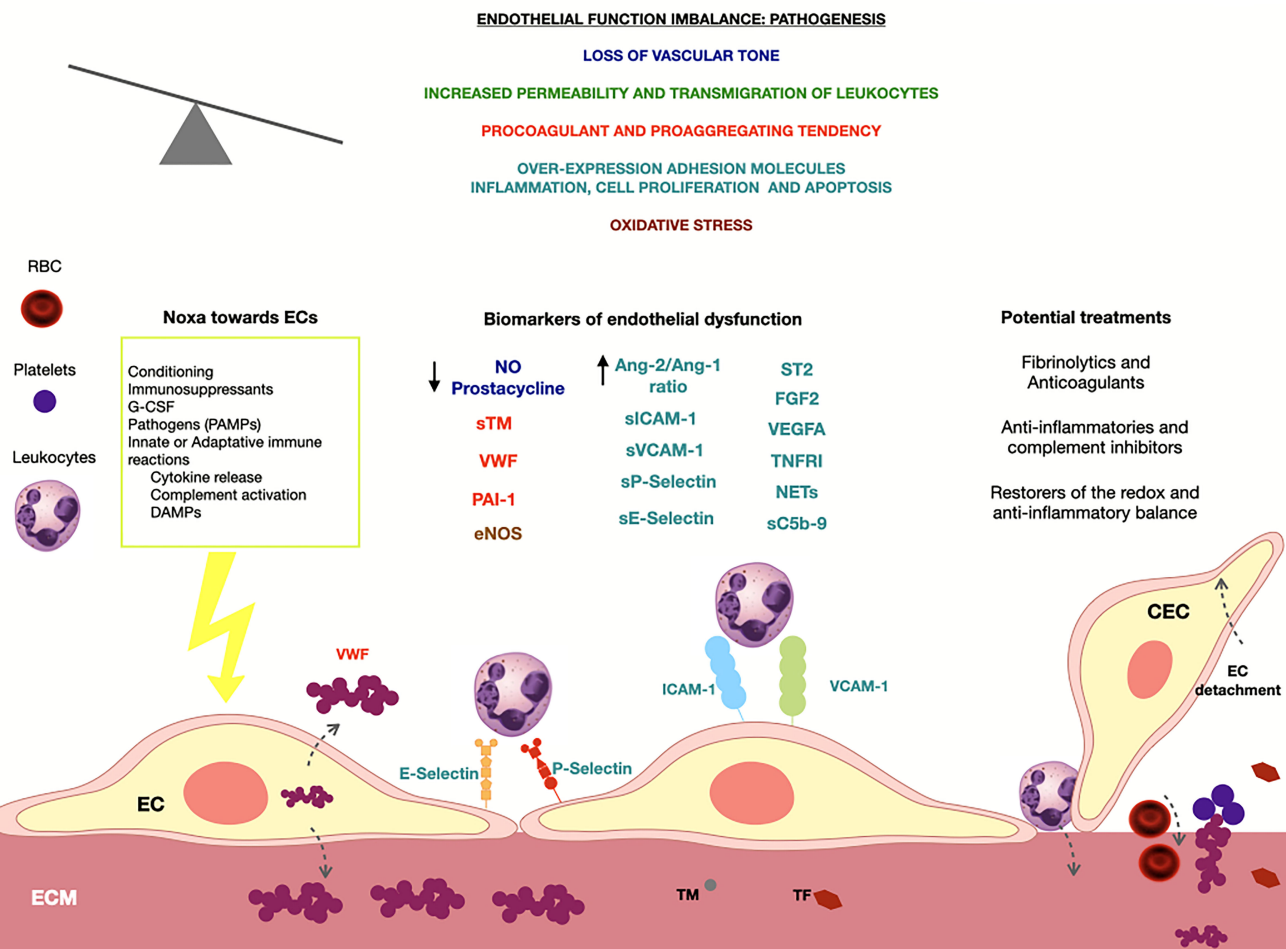
At left, the main noxa towards the endothelium in the context of the HCT are summarized. In the upper part of the figure, the principal pathways started after a loss of the endothelial-function equilibrium are described. Below, different biomarkers of endothelial dysfunction are shown and their color represents the pathway in which are involved. At right, the principal treatments developed targeting the endothelium are exposed. NO, nitric oxide; sTM, soluble thrombomodulin; VWF, von Willebrand factor; PAI-1, plasminogen activator inhibitor 1; EVs, Endothelial extracellular vesicles; CEC, Circulating endothelial cells; eNOS, endothelial nitric oxide synthase; Ang-2, angiopoietin 2; Ang-1, angiopoietin 1; sICAM-1, soluble intercellular Adhesion Molecule 1; sVCAM-1, soluble vascular cell adhesion molecule-1; ST2, soluble suppression of tumourigenicity 2; FGF2, fibroblast growth factor 2; VEGF, vascular endothelial growth factor A; TNFRI, soluble TNF receptor I; NETs, neutrophil extracellular traps; sC5b-9, soluble c5b-9 complex.

In consequence, the endothelial dysfunction occurring after HCT and derived from the mentioned stressors consists of: a) the increased synthesis of angiopoietin-2 (Ang-2), a molecule involved in the endothelial inflammation that increases its permeability, and that is upregulated over its antagonist, angiopoietin-2 (Ang-1), which has endothelial-protective properties in its counterpart (67); b) the overexpression of adhesion molecules (such as intercellular adhesion molecule 1 (ICAM-1), vascular-cell adhesion molecule 1 (VCAM), E-selectin, P-selectin), which induce leukocyte recruitment and transmigration through the endothelium (68); c) dysregulation of the vascular tone, due to the decreased synthesis of endothelial nitric oxide (NO) and prostacyclin; and d) the elevation of angiogenic molecules such as vascular endothelial growth factor A (VEGFA), fibroblast growth factor 2 (FGF2), and Ang-2, which operate through their respective receptors (VEGFR1 and VEGFR2, FGR1 and TIE-2) (69) (**Figure 1**).

### Sinusoidal obstruction syndrome

Almost thirty years ago, the first evidence of endotheliopathy as the pathophysiological substrate of SOS (70, 71), constituted a true hallmark in the HCT-therapeutics. In particular, the histological findings subjacent in SOS consisted of severe damage of the sinusoidal endothelial cells causing centrilobular coagulative necrosis, sinusoidal hemorrhage, and subendothelial fibrosis, causing portal hypertension (72). The depletion of glutathione, as a response to an acute endothelial injury, negatively affects the metabolism of some alkylating drugs used in the conditioning, potentiating their toxic effect on the ECs (73). Moreover, the obstruction of the hepatic sinusoids is caused by the overactivity of the matrix metalloproteinase (74), which increases the endothelium permeability and permits the extravasation of platelets and other blood cells into the space of Disse. The impaired production of nitric oxide (NO) by sinusoidal endothelial cells after being injured by monocrotaline was demonstrated in a mice model, where the ulterior administration of a NO-donor proved to restore the endothelial integrity and prevent SOS development (75). Furthermore, hypofibrinolysis also enhances the prothrombotic phenotype of this complication (72, 76, 77).

The growing knowledge of the pathophysiology of SOS laid the foundations for the development of its first treatment, defibrotide, focused on the protection and the re-establishment of the anti-inflammatory and anti-thrombotic properties of the endothelium (78–80). Although the role of defibrotide as a prophylactic strategy in front of HCT complications has been extensively explored (81–83), the approved indications for its use are still restricted to the treatment of severe cases of SOS with renal or pulmonary dysfunction (84, 85). Since then, endotheliopathy has been a common pathway involved in other early HCT complications characterized by an inflammatory, pro-vascular permeability and/or prothrombotic clinical presentation.

### Engraftment syndrome

ES is likely the result of a systemic endothelial damage produced by the massive release of pro-inflammatory cytokines (such as IL-2, TNF-α, IFN-γ, IL-6), and products of degranulation and oxidative metabolism of neutrophils (86, 87). In addition, the concomitant administration of G-CSF, a potent endothelial toxic (62, 88), has been observed to contribute to ES development (86, 87, 89, 90). Moreover, endothelial dysfunction has recently proven to lie beneath ES and precede its development (91).

### Capillary leak syndrome

As mentioned above, the current evidence of the endotheliopathy underlying CLS is by extrapolation with the biological data in Idiopathic Systemic CLS or Clarkson disease, which has a possible link with monoclonal gammopathies (28, 30, 92). A relationship between the administration of granulocyte macrophage colony-stimulating factors, G-CSF, and pro-inflammatory cytokines, and the development of CLS has been observed (30). In addition, the increment of circulating levels VEGF and Ang-2 documented in patients with CLS has supported the role of endotheliopathy as its pathophysiological substrate (26).

### Transplant-associated thrombotic microangiopathy

In the allogenic HCT setting, TA-TMA has been broadly ratified as an essentially vascular complication. This syndrome is characterized by affecting the renal and intestinal arterioles, mainly. Histologically, the intestinal TA-TMA shows the presence of schistocytes, fibrin, and, in severe cases, microthrombi in the intraluminal space and endothelial cell detachment (93). In the renal TA-TMA, the glomerular capillaries are also affected (94). Moreover, a tight link between TA-TMA and severe or refractory GVHD has been demonstrated in several studies, clinically and biologically (95–98).

Among all the biological pathways affecting the endothelium, the activation of the complement system seems to be the main protagonist in the TA-TMA scenario. The complement cascade is a part of both, the innate and the adaptative immune system, and can be activated by several triggers, such as residues in pathogen surfaces, deregulation of the unspecific basal activation, and the antigen-antibody union. All these pathways collide in the membrane attack complex, composed of the assembled proteins C5b-9, which binds and perforates the surface of pathogens or cells for their destruction. Moreover, products released by mobilized neutrophils, such as neutrophil extracellular traps (NETs), have a determinant role in activating the complement cascade (99, 100). NETs are double DNA strands able to trap circulating pathogens, activate the complement system, and induce a direct cytotoxic effect in the endothelium (101). Whereas the quantification of the deposit of C5b-9 on cultured endothelial cells has been demonstrated to be a sensitive tool for the functional diagnosis of other thrombotic microangiopathies, like atypical hemolytic uremic syndrome (aHUS) and severe pre-eclampsia (102, 103), its role for the diagnosis of TA-TMA is still under investigation.

### Acute graft-versus-host disease

The pathogenesis of aGvHD was firstly attributed exclusively to the T-cell-based immune alloreactivity of the graft towards the recipient’s tissues since the histology of the affected organs reveals the presence of inflammatory cellular infiltrates, mainly composed of CD3+ lymphocytes. Nevertheless, other effectors such as innate myeloid cells, damage-associated molecular patterns (DAMPs), pathogen-associated molecular patterns (PAMPs), mainly from bacterial growth, and pro-inflammatory cytokines are known today to be also involved in its development (104, 105). Based on the pathways initiating GvHD, some common with other complications after the allo-HCT, previous studies pointed to the endothelium as a centerpiece for its development (106–108). Cordes et al. recently demonstrated the presence of signs of blood-vessel apoptosis in intestinal biopsies from patients with aGvHD. Moreover, they observed, also by histological analysis, severe alterations in the endothelial microstructure and decreased expression of endothelial tight junction proteins in the organs affected by aGvHD in their murine model (109). Early angiogenesis has even been postulated as an initiator of aGvHD by enhancing the leukocyte transmigration toward the affected organs (110).

### Vascular idiopathic pneumonia syndrome/diffuse alveolar hemorrhage

Although the evidence of an infectious process is a criterion of exclusion for IPS, the presence of pathogens in the bronchoalveolar lavage in IPS patients indicates that infections might participate in IPS’s etiology and determine its prognosis (111). The lung histology from IPS patients is characterized by endothelial injury in pulmonary arterioles, seen as intravascular fibrin deposits, perivascular concentric fibrosis, and luminal thrombosis (49). The pathogenesis of DAH, specifically, is based on diffuse capillaritis caused by an intense inflammatory reaction, mediated mainly by tumor necrosis factor alpha (TNF-α), and significant apoptosis of pulmonary endothelial cells (112). In addition, he generalized loss of the integrity of the alveolar-capillary barrier leads to increased leukocyte extravasation, feedback of the inflammatory reaction, and accumulation of cells on the alveolar space (113, 114).

### Other syndromes with endotheliopathy as pathophysiological substrate

Endotheliopathy has proven to be involved in the development of the toxicities of other cellular therapies, such as cytokine release syndrome (CRS) or immune effector cell-associated neurotoxicity syndrome (ICANS) in chimeric antigen receptor (CAR) T-cell immunotherapy (115–118) although their exact pathogenesis is still under study.

## Biomarkers of endothelial damage for the diagnosis, prognostic assessment and follow-up of early HCT complications

Different biomarkers of endothelial activation and dysfunction have been found to be increased in all the post-HCT complications mentioned above. Nevertheless, most of them have been focused on evaluating the diagnosis or treatment response of SOS, TA-TMA, and GVHD, as their prevalence and clinical repercussion are higher than other post-transplant vascular endothelial complications. With this evidence, significant efforts have been made in the last years to explore whether some of these biomarkers might have a role in predicting their appearance and prognosis or in developing target EC therapies (119). However, despite the multiple efforts dedicated to the investigation and definition of biomarkers for post-transplant endothelial complications, the majority of them are cost-effective, not easily reproducible, and do not present sufficient specificity to be implemented in daily clinical practice

**Table 1** provides an overview of the potential utilities of different identified endothelial activation and dysfunction biomarkers. As reported in the previous section, the first step toward endothelial dysfunction is loss of vascular integrity and inflammatory response leading to a local increase in permeability or significant endothelium contraction, resulting in subendothelial exposure and provoking a protein “landscape” of the cell membrane. These dynamics generate the synthesis or overexpression of different adhesion or angiogenic molecules, coagulation factors, or pro-inflammatory mediators that can be harnessed as soluble biomarkers of endothelial dysfunction (67–69). In addition, endothelial damage can progress in a loss of EC integrity and shedding of endothelial cells into the bloodstream, generating a potential biomarker target of endothelial dysfunction. In particular, the presence and proportion of circulating endothelial cells (CEC) and endothelial progenitor cells (EPC) in blood correlate with vascular health homeostasis, being the presence of CEC a recognized biomarker of ongoing endothelial damage, whereas EPC could potentially evaluate vascular repair suitability (145). Nevertheless, the presence of CEC in bloodstream samples is also a dynamic phenomenon after HCT because is affected by several factors, such as the conditioning regimen, engraftment, infections and immunosuppressive treatments and this fact has to be considered when investigating the utility of these parameters as diagnostic or prognosis biomarkers for post-HCT complications (146). Lastly, endothelial cell progenitors, miRNAs, and extracellular vesicles (EV) seem to have a promising utility for the diagnostic, prediction, or targeted treatments of early post-HCT complications (122, 135, 147). However, further investigations are still needed as limited studies have been conducted on this setting.

**Table 1:** Summary of the potential uses of biomarkers of in the diagnosis, prediction, prognosis or evolution assessment of the early-HCT endothelial complications.

	Diagnostic confirmation	Prediction/Prognosis	Follow-up
**SOS**	ST2, Ang-2, L-Ficolin, HA, sVCAM-1 (120)	L-Ficolin, HA, sVCAM-1 (120)	EVs (121)
	PAI -1 Ag (72, 76, 77)	EASIX score (123)	
	EVs (121)		
	MiRNA (122)		
**ES**	TNFRI (91)	VWF, TNFRI (91)	
**CLS**	VEGF, Ang-2 (26).		
**TA-TMA**	NETs, sC5b-9 (100, 124)	ST2 (125)	sC5-b9 (131)
	Decreased haptoglobin (35)	NETs (126)	
		sC5-b9 (127)	
		Factor Ba (128–130)	
**aGVHD**	VWF, TNFRI (132)	Ang-2, sTM, HGF, IL-8 (139)	
	IL-2, IL-8, TNFRI, HGF (133)	IL-2, IL-8, TNFRI, HGF (133)	
	CEC (134)	Ang-2 (140)	
	EVs (135–138)	MAGIC score (ST2, REG3α) (141, 142)	
	miR155 (138).	ST2 (125)	
		NETs (98)	
		EVs (135–138)	
		miR155 (138)	
**Vascular-IPS/DAH**	ICAM-1, VCAM-1, eNOS (49)		
	Ang-2 (143, 144).		

SOS, sinusoidal obstructive syndrome; CLS, capillary leak syndrome; TA-TMA, transplant-associated thrombotic microangiopathy; aGvHDGVHD, acute graft versus host disease; IPH, idiopathic pneumonia syndrome; DAH, diffuse alveolar damage; ES, engraftment syndrome; ST2, soluble suppression of tumourigenicity 2; Ang-2, angiopoietin 2; HA, hyaluronic acid; VCAM-1, vascular cell adhesion molecule-1; PAI-1, plasminogen activator inhibitor 1; VEGF, vascular endothelial growth factor; NETs, neutrophil extracellular traps; sC5b-9, soluble c5b-9 complex; ICAM-1, intercellular Adhesion Molecule 1; eNOS, endothelial nitric oxide synthase; TNFRI, soluble TNF receptor I; VWF, von Willebrand factor; sTM, soluble thrombomodulin; IL-2, interleukin 2; IL-8, interleukin-8; REG3α, regenerating islet-derived 3-alpha; Factor Ba, fragment from factor B formed by the activation of the alternative pathway of the complement cascade. EVs, endothelial extracellular vesicles; miR155, microRNA-155; CEC, circulating endothelial cells; NETs, Neutrophil extracellular traps.

### Sinusoidal obstruction syndrome

The endothelial dysfunction underlying SOS has been demonstrated through different soluble biomarkers from *in vitro* and ex vivo studies. Higher circulating of coagulation factors such as Von Willebrand Factor (VWF), thrombomodulin (TM), plasminogen activator type-1 (PAI-1) together with membrane-bound intercellular adhesion molecule-1 (ICAM-1), E-selectin levels or circulating angiogenic factors, as for example, VEGF and ang-2, have been documented in patients with SOS. Based on these investigations, Aki et al. designed a biomarker panel including L-Ficolin, HA, and VCAM1 to identify patients with high-risk SOS when measured on the day of the stem cell infusion, and a second biomarker panel including circulating soluble suppressor of tumorigenicity 2 (ST2), Ang-2, L-Ficolin, HA, and VCAM1 for the diagnosis of this complication (120). Different studies have demonstrated increased levels of plasminogen activator inhibitor-1 (PAI-1), a hypofibrinolysis soluble biomarker, in patients with SOS (72, 76, 77). These results support the existence of an ongoing procoagulant and hypofibrinolytic status, suggesting a possible role for anticoagulant therapy in this setting. Higher circulating levels of PAI-1 have been documented in patients with SOS but not in those with GVHD or other liver diseases, supporting its potential use as a diagnosis marker due to its higher sensitivity (60). Moreover, the measurement of decreased PAI-1 during the first two weeks of defibrotide treatment correlated with a higher probability of presenting a complete SOS response at three months post-HCT (148).

Recent studies investigated the potential utility of miRNA or endothelial extracellular vesicles (EVs) as biomarkers in SOS (121, 122, 147). EVs are bone-marrow-derived mesenchymal stem cells circulating in peripheral blood and involved in intercellular communication by transferring proteins, lipids, and genetic material (mRNA, microRNA, lncRNA) to target cells (122). Different studies have documented that these microparticles induce angiogenesis and may repair injured endothelium by releasing paracrine mediators. Piccin et al. observed an early post-HCT increase of CD144+ EVs in plasma samples of SOS patients. Moreover, PAI-1 levels showed an increased relationship with platelet counts and were inversely correlated with EVs, and the EVs generated by the rupture of gap junctions increased in SOS patients and showed a change over time (121). Based on these results, the measurement of PAI-1 and, eventually, EVs could potentially be used for SOS diagnosis or monitoring. Although, further research is needed before universalizing these biomarkers as part of the routine diagnostic work-up for SOS, and to evaluate their potential clinical repercussion if used for targeted therapies in this setting.

### Transplant-associated thrombotic microangiopathy

In the setting of TA-TMA, NETs and soluble C5b-9 have been postulated as potential biomarkers for diagnosis confirmation (100, 124), and it might be used to foresee the development of TA-TMA (126) and of aGvHD (98). Also, the early assessment of some coagulation factors, such as VWF and TM, together with soluble vascular CAM protein 1 (sVCAM-1), or biomarkers belonging to the complement cascade, such as sC5b-9 or Factor Ba, can predict TA-TMA development (128–130) and even guide the treatment (131). Jodele et al. recently demonstrated that activated terminal complement, measured by elevated blood soluble C5b-9, alone, is a valuable indicator of reduced survival in a prospective study including 130 patients undergoing HCT with a diagnosis of TA-TMA published in 2022 A “dose effect” was observed between higher sC5b-9 levels, higher risk for developing multiorgan dysfunction syndrome, and worse outcomes. This study lastly suggests that scheduled soluble C5b-9 measurements could promptly identify patients at risk for poor outcomes and would facilitate early TA-TMA-directed therapy to prevent organ injury. Moreover, an updated TA-TMA risk algorithm incorporating laboratory biomarkers, clinical findings, and comorbid conditions was generated using this study’s findings for managing TA-TMA (127). Lastly, the measurement of haptoglobin in blood samples from TA-TMA patients has been proposed as diagnostic criteria. Moreover, recent proteomics profiling on serum performed in patients undergoing HCT has permitted the isolation of a 17 KDa haptoglobin degradation product that was differentially expressed in patients who developed TA-TMA (35). This non-invasive biomarker showed diagnostic value toward TA-TMA and could allow earlier intervention.

### Acute graft-versus-host disease

Different soluble biomarkers of endothelial dysfunction have been postulated for GVHD diagnosis. These biomarkers involve, among others, coagulation factors such as VWF or soluble thrombomodulin (sTM), circulating angiogenic factors such as VEFG or ang-2, or inflammatory cytokines such as TNFα (132). In addition, higher levels of ang-2, soluble thrombomodulin (sTM), hepatocyte growth factor (HGF), and interleukin 8 (IL-8) have been identified as potential GVHD predictive biomarkers or as predictors of corticosteroid refractoriness (139, 140). Identifying different soluble biomarkers has also permitted the design of different panels for diagnosis or prognostic stratification. Paczesny et al. developed a model composed by four biomarkers (Il-2, TNFR1, IL-8, and HGF) for the laboratory confirmation of GvHD and its prognostic stratification In the same line, and using proteomics approaches, the Mount Sinai Acute GVHD International Consortium (MAGIC) went a step further and validated an algorithm for the prediction of the risk of severe GvHD, non-relapse mortality (NRM) (141) and long-term outcomes in patients with steroid-refractory GVHD (142). Although the model was composed of two parameters meant as gastrointestinal-damage biomarkers (ST2 and REG3 α), ST2 is also produced by endothelial cells, supporting the need for assessing endothelial biomarkers in other prognostic scores. The predictive ability of MAGIC panel has demonstrated high sensitivity, and the results have been validated externally with notable success. These results have permitted the implementation of this diagnostic panel in different clinical centers. Lastly, increased pre-transplant levels of ST2 indicate a higher risk of TA-TMA (95) and, when measured at day +28, can be useful in predicting the likelihood of GvHD, together with non-relapse mortality and overall survival (125).

Different research is being conducted exploring the potential role of CEC count measurement and the quantification of circulating miRNAs and EVs in GVHD (134, 135). A relatively increased CEC count has been described by Almici et al. in patients with GvHD compared to those without this complication, and more interestingly, CEC values returned to basal pre-transplant values in responding patients. These results suggest that CEC values could eventually be treated as markers of GVHD onset or evaluate treatment response (134). Different free circulating miRNAs, such as miR155, miR146a, miR19a, miR20a, miR30, miR181, miR150, miR194, miR100, and miR518f, have been isolated in plasma/serum before HCT, two weeks after the stem cell infusion, and before the onset of GVHD, suggesting a possible prognostic use in GVHD (135). Interestingly, mir155 could potentially be used as a diagnostic biomarker among all these miRNAs, as serum up-regulation of miR155 has been observed in patients with confirmed GI-GVHD and in GVHD experimental models with mices (136). Furthermore, blocking miR155 function with a synthetic oligonucleotide complementary to miR155 has been shown to improve GVHD symptomatology in different investigations suggesting that miR155 can potentially be used as a therapeutic target in this setting. Lastly, the potential role of serum EVs as biomarkers of GVHD is also under investigation (135, 136). Lia et al. observed a significant expression change of three EVs membrane antigens in post-transplant patients before the onset of GVHD, suggesting that the routine measurement of EVs before and after HCT could have a potential utility for GVHD prediction (137). Moreover, an association between increased EVs and higher levels of circulating miR155 has been observed in both patients and animal models at the time of GVHD onset but also before starting the clinical manifestations suggesting a possible prognostic use of miRNAs together with EVs (138). Nevertheless, although the results provided by these investigations are promising, the potential utility of these biomarkers for GVHD diagnosis, prognosis, or for the design of specific targeted-treatments is not yet defined, validated, or standardized

### Additional biomarkers in early post-transplant complications

Increased plasma levels of VWF and TNFRI have been documented in patients with ES, suggesting that these biomarkers could have potential use for diagnostic confirmation. Moreover, increased levels of TNFR1 were identified on day +5 after auto-HCT in patients who after developed this complication suggesting that TNFR1 could also be a useful biomarker for ES prediction (91). Moreover, levels of circulating VEGF and Ang-2 have also been found to be elevated in CLS (26), and higher levels of the endothelial-damage biomarkers ICAM-1, VCAM-1, eNOS (49), and Ang-2 have been described in patients with IPS/DAH (143, 144).

Lastly, the Endothelial Activation and Stress Index (EASIX) was developed as a biomarker-based laboratory formula defined as creatinine (mg/dL) x lactate dehydrogenase (LDH; U/L)/platelets (x 109/L) to predict mortality in patients with aGVHD (149). EASIX, when measured at different time points before and after the stem cell infusion, has additionally been shown to be useful for the prediction of mortality after allo-HCT (150), TA-TMA (150), ICU admission (151), and SOS (123). Moreover, a modified version of EASIX, which substitutes creatinine for C reactive protein, demonstrated predictive utility for CRS and ICANs in CAR-T cell patients (152, 153). Although the use of EASIX is not yet standardized, its implementation in clinical practice can potentially simplify the prediction of different vascular ECs after HCT, eventually permitting the arrangement of additional diagnostic tests or preemptive interventions.

## The endothelium as a therapeutic target in HCT

Considering the role of the endothelium in the pathophysiology of these early post-HCT complications, different treatment strategies focused on targeting EC dysfunction have been approved or are under investigation with promising results (7, 9).

### Defibrotide

Defibrotide has been shown to reduce EC activation and to exert a fibrinolytic effect through the enhancement of tissue plasminogen activator (t-PA) and thrombomodulin synthesis and by decreasing the expression of PAI-1 (79, 85). Furthermore, its efficacy was proved in a controlled, phase 3 trial, where, compared with historical control cases, the use of defibrotide for the treatment of post-HCT SOS with multiorgan failure increased the probability of day +100 post-transplant survival from 25% to 38%. Consecutively, defibrotide was demonstrated to be effective for treating SOS without MOD, and its use resulted in a day +100 probability of survival of 56% (154). Secondary to its efficacy, using defibrotide as primary or secondary prophylaxis for SOS is considered in high-risk patients (19, 155). Furthermore, although its use in this context is not yet approved, prophylactic defibrotide effectively decreases the incidence of TA-TMA and GvHD in pediatric patients (155–157).

### Other anticoagulant and fibrinolytic agents

Heparin, whose anti-inflammatory effects have also been demonstrated (158), has been shown to decrease the incidence of SOS without significant bleeding complications when used at low doses (159, 160). Other agents with anticoagulant properties, such as protein C concentrates (161) and recombinant thrombomodulin (162, 163), might have a role in the prevention and treatment of SOS. Recombinant tissue plasminogen activator (Rt-PA) has also been demonstrated to effectively treat SOS (164, 165), although a risk-benefit balance must be considered before its use.

### Anti-inflammatory agents

High-dose corticoids are considered the first line of treatment for acute GvHD and other endothelial-related post-transplant complications such as ES, IPS, and DAH (9). Corticosteroids have anti‐inflammatory effects that may mitigate endothelial damage and are commonly used to manage the pro‐inflammatory state associated with endothelial‐related HCT complications (46, 49). The use of the anti‐TNFα agent etanercept to restore EC function and decrease inflammatory chemokine expression to treat IPS has been explored by different investigators (166). However, reported data has been inconsistent, and the reduced sample size of patients has limited the conclusions included in the studies. Nevertheless, hypotheses are sustained that its use with corticosteroids could potentially increase post-transplant outcomes in patients who did not require positive pressure ventilation at the time of diagnosis (166).

### Complement inhibitors

The complement protein C5 antibody eculizumab is an effective therapeutic strategy for post-transplant patients with high-risk TA-TMA. Its use has been associated with high rates of clinical response and an improvement in 1-year overall survival (40, 131). The role of other inhibitors of the complement cascade, such as ravulizumab, coversin, pegcetacoplan, crovalimab, avacopan, iptacopan, danicopan, BCX9930, and AMY-101, is currently being explored in the setting of TA-TMA (167).

### Re-establishers of the endothelial redox and the anti-inflammatory balance

N-acetylcysteine (NAC) is an antioxidant agent with an excellent safety profile, which has been demonstrated to be effective in treating SOS in a limited cohort of pediatric patients (168). In addition, other agents known as endothelial stabilizers, such as Ang-1 (169) and nitric oxide-prodrugs (75), sildenafil (109) and statins (170, 171) demonstrated to restore the physiological endothelial properties when administered exogenously in murine models or *in vitro*. Nevertheless, their effects in the specific context of HCT complications still need to be explored.

## Conclusions and future perspectives

There is growing evidence pointing to the endothelium and other linked pathways as pathophysiological substrates of the main HCT complications. Panels composed of endothelial-damage biomarkers are being developed to early predict these complications, their risk stratification, and the ulterior follow-up. In addition, multiple therapeutic and prophylactic strategies oriented to endothelial protection are being proposed, and their impact on the incidence of complications and non-relapse mortality.

The use of non-invasive biomarkers for detecting and diagnosing early post-HCT endothelial complications is a promising field of research. However, the lack of consistency among studies, probably secondary to patient`s heterogenicity and discrepancies in transplant techniques, difficult to implement these biomarkers in clinical practice. Moreover, the lack of validation techniques and additional costs derived from measuring certain biomarkers are also difficult on a daily basis. Nevertheless, based on the extensive data on endothelial circulating biomarkers’ role as practical tools for the mentioned purposes, further efforts must be done to implement these techniques in clinical practice.

## Author contributions

ABM-C and MQS wrote the manuscript. GE, MD-R and ABM-C designed the figure. ABM-C summarized the information in the table, and all the authors contributed to the review and editing of the final manuscript. All authors contributed to the article and approved the submitted version.

## Funding

This contribution has been partially supported by Fundació Clínic, Barcelona (HCB/2020/0401), Jazz Pharmaceuticals Plc (IST-16-10355), German José Carreras Leukaemia Foundation (03R/2019), Instituto de Salud Carlos III from Spanish Government (PI19/00888), Fundació La Marató de TV3 (202026–10), Bristol Myers-Squibb (ERISTA 15) and Generalitat de Catalunya (2017-SGR671 and CERCA Program). The funder was not involved in the study design, collection, analysis, interpretation of data, the writing of this article or the decision to submit it for publication.

## Conflict of interest

The authors declare that the research was conducted in the absence of any commercial or financial relationships that could be construed as a potential conflict of interest.

## Publisher’s note

All claims expressed in this article are solely those of the authors and do not necessarily represent those of their affiliated organizations, or those of the publisher, the editors and the reviewers. Any product that may be evaluated in this article, or claim that may be made by its manufacturer, is not guaranteed or endorsed by the publisher.
